# Characterization of Monoclonal Antibodies against HA Protein of H1N1 Swine Influenza Virus and Protective Efficacy against H1 Viruses in Mice

**DOI:** 10.3390/v9080209

**Published:** 2017-08-08

**Authors:** Yun Liu, Hongtao Li, Yujia Xue, Shuang Zhao, Chenxi Li, Liandong Qu, Yun Zhang, Ming Liu

**Affiliations:** State Key Laboratory of Veterinary Biotechnology, Harbin Veterinary Research Institute of Chinese Academy of Agricultural Sciences, Harbin 150001, China; 15624955874@163.com (Y.L.); hongtaoli05@126.com (H.L.); xueyj@126.com (Y.X.); zhaos01@126.com (S.Z.); lichenxihsy@126.com (C.L.); qld@hvri.ac.cn (L.Q.)

**Keywords:** swine influenza virus, HA protein, monoclonal antibodies, protection efficacy

## Abstract

H1N1 swine influenza viruses (SIV) are prevalent in pigs globally, and occasionally emerge in humans, which raises concern about their pandemic threats. To stimulate hemagglutination (HA) of A/Swine/Guangdong/LM/2004 (H1N1) (SW/GD/04) antibody response, eukaryotic expression plasmid pCI-neo-HA was constructed and used as an immunogen to prepare monoclonal antibodies (mAbs). Five mAbs (designed 8C4, 8C6, 9D6, 8A4, and 8B1) against HA protein were obtained and characterized. Western blot showed that the 70 kDa HA protein could be detected by all mAbs in MDCK cells infected with SW/GD/04. Three mAbs—8C4, 8C6, and 9D6—have hemagglutination inhibition (HI) and neutralization test (NT) activities, and 8C6 induces the highest HI and NT titers. The protection efficacy of 8C6 was investigated in BALB/c mice challenged with homologous or heterologous strains of the H1 subtype SIV. The results indicate that mAb 8C6 protected the mice from viral infections, especially the homologous strain, which was clearly demonstrated by the body weight changes and reduction of viral load. Thus, our findings document for the first time that mAb 8C6 might be of potential therapeutic value for H1 subtype SIV infection.

## 1. Introduction

Eurasian H1N1 swine influenza virus (SIV) was first reported in pigs in 1979 [1] and then circulated in the European pig population [2]. Eurasian H1N1 SIV was first reported in China in 1993 and has occurred frequently in pigs [3,4]. Since 2009, a pandemic H1N1 SIV was detected in Mexico, and then spread rapidly to other countries, such as China, Italy, the United States, and Canada [3,5,6,7,8]. Three subtypes of SIV—H1N1, H3N2, and H1N2—have been reported in pigs, globally [9].

Pigs, which serve as ‘mixing vessels’ because of their susceptibility to infection by both human and avian influenza viruses, may be a pandemic threat to public health [10]. Sporadic human infection with the Eurasian H1N1 SIV has emerged in Europe and China [11,12,13]. Vaccination is a primary and effective measure for controlling SIV infection [14,15], but it might have some restrictions. For example, vaccinations may not be effective in preventing against diverse viral strains, manifesting as less immunogenic, or acting with inadequate speed, to combat newly-emerging seasonal or potentially pandemic strains [16]. Other approaches, including viral culture in mammalian or insect cells, have been suggested to produce pandemic or seasonal influenza vaccines [17,18], but the low levels of expressed proteins, or the unknown risks of antigens in cells, are obstacles in combating pandemics [19]. Therefore, there is an urgent need to develop an alternative rapid measure to cope with the requests of a pandemic [20]. For example, passive immunization by delivering specific antibodies to a recipient could protect animals from infection [21]. Neutralizing monoclonal antibodies (mAbs) against virus function either by inhibiting virus attachment to, or membrane fusion with, the susceptible cells [22]. Studies have proved that mAbs could be used as an effective and preventive treatment against influenza virus infection [23,24,25,26,27]. However, until now, there are no effective neutralizing mAbs available in preventing or controlling H1N1 SIV infection.

Production of functional antibodies is highly dependent on the structural integrity of the proteins [28,29,30]. Traditional protein-based immunization has difficulty in generating mAbs against conformation-sensitive targets. DNA-based immunization can solve these problems because native proteins can be expressed in vivo when they are delivered in the form of DNA as an immunogen, which does not require the process of protein production or purification. Furthermore, the correct conformation of proteins is critical for the induction of functional active antibodies, yet these sensitive structures tend to be damaged during the in vitro protein production process. Expressing intact immunogens in vivo by DNA-based immunization appears to be the best approach for inducing mAbs with the desired biological activities [31]. Herein, a eukaryotic expression plasmid (pCI-neo-HA) was constructed and used as the immunogen to prepare mAbs against hemagglutination (HA) protein of H1 subtype swine virus. We prepared and characterized five mAbs and then evaluated 8C6 protective efficacy in mice against infection with homologous and heterologous H1 subtype viruses.

## 2. Materials and Methods

### 2.1. Ethics Statement

All experiments and procedures involving animals were approved by the Animal Welfare and Ethical Censor Committee at Harbin Veterinary Research Institute (HVRI). All animal experiments in this study were approved by the Animal Ethics Committee of the HVRI of the Chinese Academy of Agricultural Sciences with license SYXK (Heilongjiang) 2011022.

### 2.2. Virus Strains

Two viral strains of the H1N1 were used: A/Swine/Guangdong/LM/2004 (SW/GD/04) (H1N1) and A/Swine/Harbin/2009 (SW/HRB/09) (H1N1). The viruses were propagated in 10-day-old specific-pathogen free (SPF) embryonated chicken eggs or in Madin–Darby canine kidney (MDCK) cells and stored at −70 °C before use. MDCK cells were cultured in Dulbecco’s modified essential medium (DMEM) containing 10% (*v*/*v*) fetal bovine serum (Hyclone, UT, USA) and incubated at 37 °C and in a 5% (*v*/*v*) CO_2_ atmosphere.

### 2.3. Fifty-Percent Tissue Culture Infective Dose (TCID50) Assays

A monolayer of MDCK cells in 96-well plates were inoculated with serial dilutions of the virus strains (each dilution with five replicates). The cytopathic effect (CPE) was observed daily and the number of wells showing more than 50% pathological changes were recorded. TCID50 titers were calculated as described previously [32]. Each dilution was done in five repetitions.

### 2.4. Construction of Gene Expression Plasmids

Viral RNA was extracted from SW/GD/2004 and SW/HRB/09 allantoic fluids by using a viral RNA extraction kit (Qiagen, Shanghai, China). Virus-specific cDNAs were obtained by using influenza universal reverse transcription primer uni-12:5′-AGCAAAAGCAGG-3′ with the AMV reverse transcriptase (TaKaRa, Dalian, China). The HA genes were amplified by using HA gene-specific primers, cloned into the pMD18-T vector (TaKaRa, Dalian, China), and then sequenced by using an ABI PRISM 3700 DNA Analyzer (Applied Biosystems, Shanghai Invitrogen, China). Full-length HA was cloned into pCI-neo using specific primers, which introduced a *Nhe* I/*Xho* I restriction site. The resulting plasmid pCIneo-HA was purified using a Qiaminiprep kit (Qiagen) as per the manufacturer’s protocols. The extracted plasmid was identified by using a double digest of *Nhe* I and *Xho* I (New England Biolabs, Whitby, ON, Canada). Recombinant plasmids were transformed into TOP10 competent cells. Colonies were screened via PCR to confirm insertion of the gene segments. The plasmid sequencing was conducted by using an ABI 3730 DNA automatic sequencer.

### 2.5. Hybridomas Antibody Production

Plasmid DNA, pCIneo-HA was used as the immunogen for development of mAbs in this study. Briefly, five-week-old female BALB/C mice were injected intramuscularly (i.m.) with 50 µg of plasmid DNA in sterile phosphate buffered saline (PBS), pH 7.4. The mice received two boosts of 100 µg of plasmid DNA at a three-week interval. The mice spleens were collected aseptically by using euthanized anesthesia techniques. mAbs were produced using techniques similar to that described previously [33,34,35]. Splenocytes were fused with SP2/0-Ag14 myeloma cells. Hybridoma cell lines secreting antibodies against HA were screened for HA antibodies in an indirect ELISA and subcloned at least three times by a limiting dilution method. Ascitic fluids were prepared with the cloned hybridoma in BALB/c mice. Isotypes of the obtained mAbs were determined by using a mouse immunoglobulin isotyping kit (Zymed Laboratories, Inc., USA) according to the manufacturer’s instruction.

### 2.6. Hemagglutination Inhibition Test

The hemagglutination inhibition (HI) test was performed to evaluate mAbs reactivity against SW/GD/04 as described previously [36,37]. Briefly, 25 μL of serial two-fold dilutions of the purified ascetic fluids were mixed with four HA units of virus in hemagglutination plates and incubated at 37 °C for 30 min. Then, 25 μL of 1% chicken red blood cells were added to each well and incubated for another 30 min. To rule out non-specific inhibition, the ascetic fluids produced from the injection of SP2/0 myeloma cells were used as a negative control. The HI titer was expressed as the reciprocal of the highest ascetic dilution that completely inhibited hemagglutination of four HA units of the virus [37]. Each mAb was repeated three times.

### 2.7. Neutralization Test

The Neutralization Test (NT) was performed using 96-well plates. Mixtures of two-fold serial dilutions of each mAb and virus suspension containing 100 TCID50 of SW/GD/04 were incubated for 1 h at 37 °C and used to inoculate MDCK cells. One hundred microliters (100 µL) of DMEM was added to each well and incubated at 37 °C for three days. The CPE was observed every 24 h. Neutralization titers are presented as reciprocals of the highest antibody dilution, causing a reduction of the virus over 50%. Each dilution was repeated five times.

### 2.8. Western Blot Assay

To examine whether anti-HA mAbs recognize the HA protein, Western blot was used to examine the binding ability of mAbs to HA proteins. Approximately 1 μg of purified SW/GD/04 virus was subjected to 10% SDS-PAGE or native PAGE and then transferred to nitrocellulose membranes. The membranes were probed with different mAbs, followed by a secondary HRP-conjugated goat anti-mouse antibody (KPL, Gaithersburg, MD, USA). The ascetic fluids produced from the injection of SP2/0 myeloma cells were used as a negative control.

### 2.9. Detection of Native HA Protein by Immunohistochemistry (IH) Assay

MDCK cells were infected with the SW/GD/04 strain (at multiplicity of infection (MOI) 10) and incubated at 37 °C for 24 h. After incubations, the monolayers were washed twice with PBS and fixed in methanol at −20 °C for 30 min. The cells were then incubated separately with different mAbs for 1 h at 37 °C or with negative ascetic fluids. Bound antibodies were processed for immunoperoxidase staining by using alkaline phosphatase-labeled mouse IgG (1:500 dilutions). After extensive washing with PBS, peroxidase activity was revealed by incubation with 0.03% Nitro blue tetrazolium chloride(NBT)/5-bromo-4-chloro-3-indolyl phosphate (BCIP), 0.006% H_2_O_2_ in PBS for 5 min.

### 2.10. Cross-Protection by mAb 8C6

mAb 8C6 was selected to evaluate its protective efficacy for H1N1 SIV because of its high HI and NT titers. A total of 40 six-week-old SPF BALB/c mice were used to evaluate the protective efficacy of mAb 8C6. Mice (*n* = 8) were pretreated intramuscularly with mAb 8C6 (two group) or negative ascetic fluids (two group) at a dose of 20 µg per mg of mouse body weight before the viral challenge [27,38]. The remaining group of mice (*n* = 8) pretreated intramuscularly with negative ascetic fluids were used as normal controls. After 24 h, mice were intranasally challenged with homologous virus SW/GD/04 or heterologous virus SW/HRB/09, respectively. Mice were monitored for weight changes and clinical symptoms for two weeks at two-day intervals. Three mice per subgroup were euthanized on day 3 post-challenge (p.c.) and samples (including the nasal turbinate, lung, spleen, and kidney) were collected for virus titration in eggs. The remaining five mice per group were observed daily for weight changes or clinical signs of infection.

## 3. Results

### 3.1. pCI-Neo-HA Construction and HA Sequence Analysis

**Figure 1:**
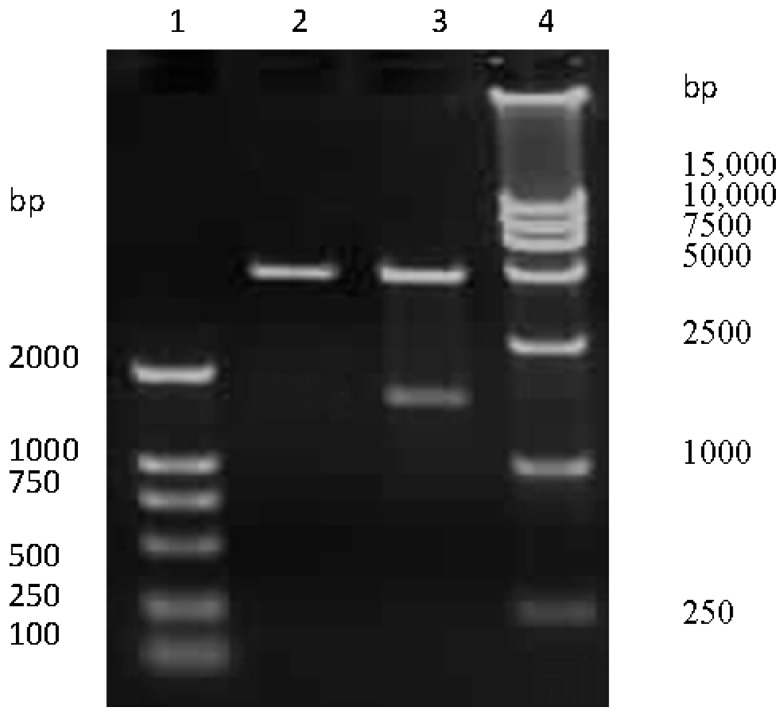
The recombinant pCI-neo-HA identification by Nhe I/Xho I digestion. Lane 1 and 4: DNA molecular weight marker; lane 2: empty pCI-neo plasmid; lane 3: pCI-neo-HA plasmid digested with Nhe I and Xho I.

The HA-encoding gene of SW/GD/04 was amplified and cloned into pCI-neo vector. To confirm the recombinant plasmids were correct, pCI-neo-HA were digested by Nhe I/Xho I restriction analysis, and then for nucleotide sequencing. Electrophoresis results revealed that pCI-neo-HA plasmids were digested into two fragments, which were consistent with the sizes of the HA-encoding gene and pCI-neo vector, respectively (Figure 1). Sequencing results confirmed that the HA-encoding gene was successfully cloned into the pCI-neo vector.

### 3.2. Production and General Characterization of mAbs

**Figure 2:**
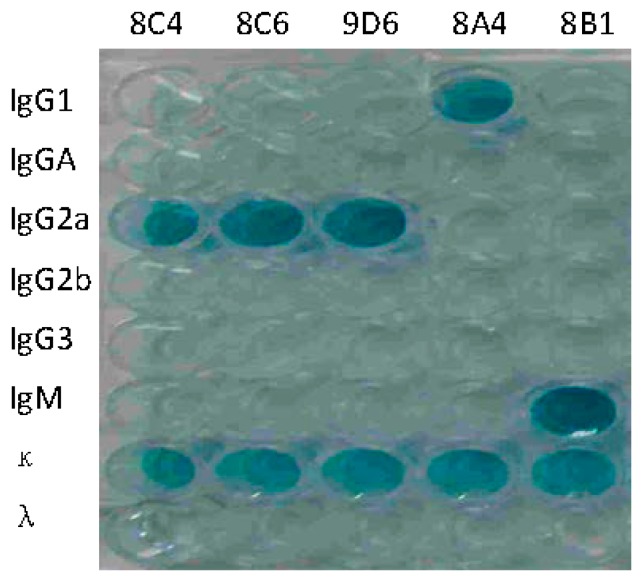
Isotype identification of prepared monoclonal antibodies (mAbs). mAbs 8C4, 8C6, 9D6, 8A4, and 8B1 are labeled on the top of the plates.

The hybridoma cell lines secreting anti-HA antibodies were screened by ELISA. Five mAbs against HA were selected for subcloning at least three times. Positive hybridomas were used to produce mAbs in mice and the obtained ascitic fluids were collected for characterization. The isotypes of mAbs were IgG1 (8A4), IgG2a (8C4, 8C6, and 9D6), and IgM (8B1), respectively (Figure 2). Concentrations of immunoglobulin ranged from 8.5 to 265.8 μg/mL. ELISA, HI, and NT titers of the five mAbs were determined. HI and NT indicated that mAbs 8C4, 8C6, and 9D6 had both HI and neutralization activities, and 8C6 had the highest HI and NT titers. mAbs 8A4 and 8B1 showed no HI and neutralization activity, but 8B1 showed the highest ELISA activity (Table 1).

### 3.3. Detection of Native HA Protein by Immunohistochemistry Assay

**Figure 3:**
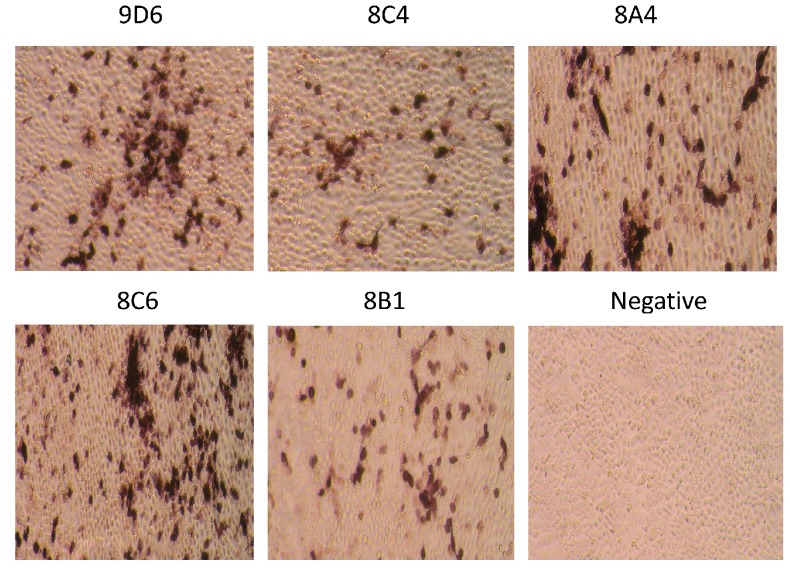
Detection of hemagglutination (HA) protein from A/Swine/Guangdong/LM/2004 (SW/GD/04) infected MDCK cells with mAbs 9D6, 8C4, 8A4, 8C6, and 8B1 by immunohistochemistry (IH). Non-infected MDCK cells are used as a negative control. Magnification: 400×.

SW/GD/04-infected MDCK cells were used to assess whether the obtained mAbs recognize the native-form HA protein by immunohistochemistry (IH) assays. Five mAbs showed strong specific reactions to SW/GD/04-infected MDCK cells, whereas no immuno-reactivity was observed in normal MDCK cells (as the negative control) (Figure 3). The black color signals were visualized in SW/GD/04-infected cells. This indicated that all mAbs were able to detect the native-form HA protein in SW/GD/04-infected cells.

### 3.4. Detection of HA Protein by Western Blot

**Figure 4:**
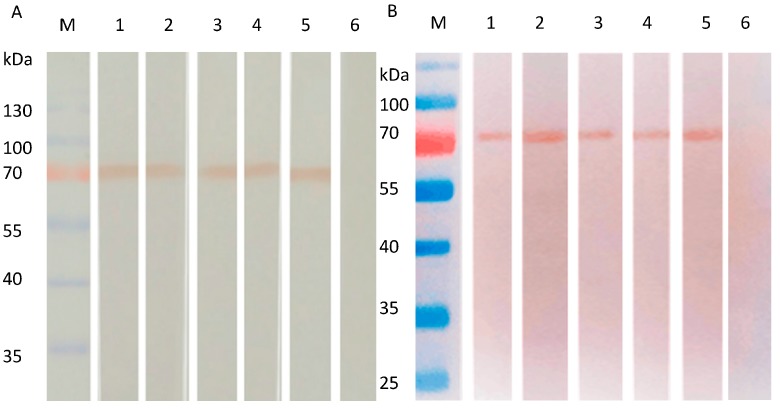
Reactivity of mAbs to HA proteins of SW/GD/04 infected MDCK cells analyzed by Western blotting. HA proteins of SW/GD/04 infected MDCK cells run in SDS-PAGE (**A**) or native PAGE gel (**B**), and then transferred membranes were detected with mAbs by Western blotting. Lane M: protein molecular weight marker; lanes 1, 2, 3, 4, and 5: SW/GD/04-infected MDCK cells run in SDS-PAGE detected by mAbs 8C4, 8C6, 9D6, 8A4, and 8B1, respectively; lane 6: represents mock-infected cells detected by mAbs.

SW/GD/04-infected MDCK cells were used to assess whether the obtained mAbs recognize the native-form or the denatured-form of HA protein by Western blot. mAbs 8C4, 8C6, 9D6, 8A4, and 8B1 reacted strongly with the 70 kDa HA proteins of SW/GD/04-infected MDCK cells in both denatured (Figure 4A) and native forms (Figure 4B), suggesting mAbs might recognize linear epitopes. Non-infected MDCK cells showed no reaction.

### 3.5. Protective Efficacy of the mAb Treatment Prior to Virus Infection

**Figure 5:**
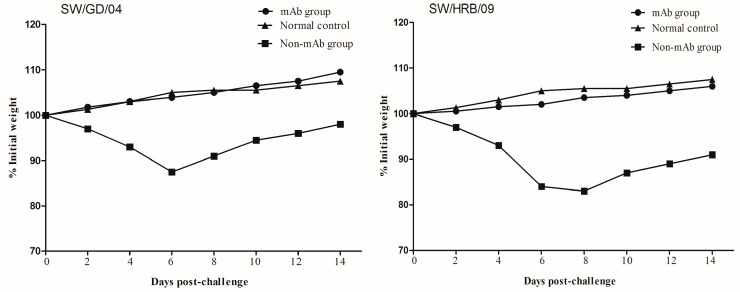
Weight changes in mice after challenge with SW/GD/04 and A/Swine/Harbin/2009 (SW/HRB/09) viruses. Groups of treated and untreated mice were intranasally inoculated with 10^6^ EID50 of SW/GD/04 and SW/HRB/09 24 h after the mAb 8C6 treatment. The body weights of each mouse were monitored at two-day intervals for two weeks. Values indicate the mean weight changes of all of the mice in each group after virus challenge.

The protective efficacy of the mAb 8C6 was evaluated in mice with two different H1N1 strains (the HA gene of SW/GD/04 showed 90% identities to that of SW/HRB/09). Body weight changes were monitored at two-day intervals in both mouse and virus titers (each titer was calculated by five repetitions) in lung, nasal turbinates, spleen, and kidney were detected on day 3 after the challenge. Compared with the normal control mice, all mAb 8C6 pre-treated mice showed no obvious clinical symptoms after challenge. When challenged with the homologous virus SW/GD/04, no weight changes were observed in the mAb 8C6-treated mice, whereas weight loss in the non-mAb-treated mice was 12.3%. Moreover, compared with mice in the normal control group, there was 15.7% weight loss in mice challenged with the heterologous SW/HRB/09 virus (Table 2, Figure 5). Moreover, there was less weight loss (1.5%) in the mAb-8C6-treated mice following challenge with heterologous SW/HRB/09.

Virus replication in mice was detected on day 3 after the challenge. In the mAb 8C6 pre-treated group, there was no detectable virus in the lung/nasal/spleen/kidney of mice following challenge with SW/GD/04. In the non-mAb-treated group, the challenge SW/GD/04 virus replicated to mean titers of 3.29 log10 EID50 in the nasal turbinates and 4.88 log10 EID50 in the lungs (Table 2). By contrast, in the mAb 8C6 pre-treated group, when challenged with SW/HRB/09, two of three mice revealed detectable virus replication in the lungs and one mouse displayed detectable virus replication in the nasal turbinates with mean titers of 1.65 and 1.36 log10 EID50, respectively, whereas the virus was detectable in all non-mAb-treated mice with titers of 5.12 and 4.39 log10 EID50 in the lungs and nasal turbinates, respectively. No virus was detected in the spleen/kidney of mAb 8C6 pre-treated or non-mAb-treated mice after challenge with SW/GD/04 or SW/HRB/09.

## 4. Discussion

Antibody-mediated passive immunity can provide protection against invading pathogens [38]. Since there is no commercial vaccine available for H1N1 swine influenza virus in China, it is important to develop other passive immunotherapeutics to prevent or control H1 subtype SIV infection in pigs. The HA glycoprotein induces the neutralizing antibodies that provide immediate acquired immunity to influenza viruses. Therefore, the generation of neutralizing mAbs against antigenic sites on the HA glycoprotein is regarded as a criterion for evaluating immunity to influenza viruses and is believed to constitute the main correlate of protection [26,27].

In this study, we developed five mAbs against HA protein of SW/GD/04 with a eukaryotic recombinant plasmid pCI-neo-HA as an immunogen in mice. Since HI and neutralization tests demonstrated that mAbs 8C4, 8C6, and 9D6 both had HI and neutralization activities, we may speculate that this neutralization activity resulted from correct folding of the HA protein. IH assay showed that mAbs reacted with the HA protein of SW/GD/04 (H1N1)-infected MDCK cells, indicating that mAbs were H1-specific. Western blot showed that mAbs recognized the 70 kDa HA protein, 6 kDa larger than the expected 64 kDa HA protein, indicating that HA might be glycosylated.

Immunization with anti-HA antibodies is capable of conferring protection against influenza virus infection in both animals and humans. Since mAb 8C6 has the highest HI and NT titers, we examined the protective efficacy in mice by challenging homologous and heterologous strains of H1N1 SIV (SW/GD/04 and SW/HRB/09). Considering H1N1 SIV isolates were not lethal to mice, we investigated the mAb protection efficacy by body weights and detectable virus titers from lungs, spleen, kidney, and nasal turbinates three days after challenge. These data confirm that one-dose pretreatment of mice with mAb 8C6 is sufficient to provide complete protection against homologous SW/GD/04 H1N1 SIV infection.

In this study, we also evaluated the ability of mAb 8C6 to protect mice from challenge with the pandemic 2009/H1N1-like virus (the heterologous strain SW/HRB/09). Compared with normal control mice, the replication of the challenge virus in the mAb-treated mice was also inhibited in the lungs and nasal turbinates. These results demonstrate that the treatment of mice with mAb 8C6 also limits the susceptibility of the mice to infection with H1N1 SIV including the HA gene of a recent EA-lineage virus, suggesting that broad cross-protection might be conferred against the pandemic 2009/H1N1 virus. These results suggest that mAb 8C6 could provide a complete protection against the homolog and inhibit disease development caused by heterologous H1N1. The mAb 8C6 is, therefore, a promising treatment for H1 swine influenza virus infection.

Sequence alignments indicated that the HA protein of SW/GD/04 showed 90% homology to that of the SW/HRB/09 virus used herein for challenge studies. The significance of this difference for cross-protection remains unknown. Further studies mapping the epitope of this mAb should be conducted to understand the antigenic properties of the H1N1 virus, which might contribute to the prevention and control of the H1N1 virus.

## Figures and Tables

**Table 1:** Characterization of seven mAbs direct against swine/GD/LM/04.

mAb	Titers of ELISA	Titers of HI	Titers of NT
8C4	640,000	320	10^2.83^
8C6	256,000	256,000	10^6.4^
9D6	160,000	512	10^5.8^
8A4	256,000	0	0
8B1	1,024,000	0	0

ELISA: Enzyme linked immune sorbent assay; HI: Hemagglutination inhibition test; NT: Neutralization test.

**Table 2:** Protection of mice by the mAb 8C6.

Challenge Viruses			Weight-Loss (%) at 14 dpi			Virus Titer (log10 EID50/mL) at 3 dpi		
	8C6-treated	Untreated	Lung Nasal Spleen/Kidney			Lung Nasal Spleen/Kidney		
			8C6-treated			Non-mAb-treated		
SW/GD//04	–	12.3	<0.5	<0.5	<0.5	4.88	3.29	<0.5
SW/HRB/09	1.50	15.7	1.65	1.36	<0.5	5.12	4.39	<0.5

<0.5 Indicates that no virus was detected from the undiluted sample. Each titer was calculated by five repetitions. dpi: days post-infection.

## References

[1] Pensaert M., Ottis K., Vandeputte J., Kaplan M.M., Bachmann P.A. (1981). Evidence for the natural transmission of influenza A virus from wild ducts to swine and its potential importance for man. Bull. World Health Organ..

[2] Brown I.H., Done S.H., Spencer Y.I., Cooley W.A., Harris P.A., Alexander D.J. (1993). Pathogenicity of a swine influenza H1N1 virus antigenically distinguishable from classical and European strains. Vet. Rec..

[3] Guan Y., Shortridge K.F., Krauss S., Li P.H., Kawaoka Y., Webster R.G. (1996). Emergence of avian H1N1 influenza viruses in pigs in China. J. Virol..

[4] Yang H., Chen Y., Qiao C., He X., Zhou H., Sun Y., Yin H., Meng S., Liu L., Zhang Q. (2016). Prevalence, genetics, and transmissibility in ferrets of Eurasian avian-like H1N1 swine influenza viruses. Proc. Natl. Acad. Sci. USA.

[5] Moreno A., Di Trani L., Alborali L., Vaccari G., Barbieri I., Falcone E., Sozzi E., Puzelli S., Ferri G., Cordioli P. (2010). First Pandemic H1N1 Outbreak from a Pig Farm in Italy. Open Virol. J..

[6] Ducatez M.F., Hause B., Stigger-Rosser E., Darnell D., Corzo C., Juleen K., Simonson R., Brockwell-Staats C., Rubrum A., Wang D. (2011). Multiple reassortment between pandemic (H1N1) 2009 and endemic influenza viruses in pigs, United States. Emerg. Infect. Dis..

[7] Ali A., Khatri M., Wang L., Saif Y.M., Lee C.W. (2012). Identification of swine H1N2/pandemic H1N1 reassortant influenza virus in pigs, United States. Vet. Microbiol..

[8] Grgic H., Costa M., Friendship R.M., Carman S., Nagy E., Poljak Z. (2015). Genetic Characterization of H1N1 and H1N2 Influenza A Viruses Circulating in Ontario Pigs in 2012. PLoS ONE.

[9] Qiao C.L., Liu L., Yang H., Chen Y., Xu H., Chen H.L. (2014). Novel triple reassortant H1N2 influenza viruses bearing six internal genes of the pandemic 2009/H1N1 influenza virus were detected in pigs in China. J. Clin. Virol..

[10] Ito T., Couceiro J.N., Kelm S., Baum L.G., Krauss S., Castrucci M.R., Donatelli I., Kida H., Paulson J.C., Webster R.G. (1998). Molecular basis for the generation in pigs of influenza A viruses with pandemic potential. J. Virol..

[11] De Jong J.C., Paccaud M.F., de Ronde-Verloop F.M., Huffels N.H., Verwei C., Weijers T.F., Bangma P.J., van Kregten E., Kerckhaert J.A., Wicki F. (1988). Isolation of swine-like influenza A (H1N1) viruses from man in Switzerland and the Netherlands. Ann. Inst. Pasteur Virol..

[12] Yang H., Qiao C., Tang X., Chen Y., Xin X., Chen H. (2012). Human Infection from Avian like Influenza A (H1N1) Viruses in Pigs, China. Emerg. Infect. Dis..

[13] Wang D.Y., Qi S.X., Li X.Y., Guo J.F., Tan M.J., Han G.Y., Liu Y.F., Lan Y., Yang L., Huang W.J. (2013). Human infection with Eurasian avian-like influenza A(H1N1) virus, China. Emerg. Infect. Dis..

[14] Dürrwald R., Krumbholz A., Baumgarte S., Schlegel M., Vahlenkamp T.W., Selbitz H.J., Wutzler P., Zell R. (2010). Swine influenza A vaccines, pandemic (H1N1) 2009 virus, and cross reactivity. Emerg. Infect. Dis..

[15] Sui J.Y., Yang D.W., Qiao C.L., Xu H.Y., Xu B.F., Wu Y.P., Yang H.L., Chen Y., Chen H.L. (2016). Protective efficacy of an inactivated Eurasian avian-like H1N1 swine influenza vaccine against homologous H1N1 and heterologous H1N1 and H1N2 viruses in mice. Vaccine.

[16] Biesova Z., Miller M.A., Schneerson R., Shiloach J., Green K.Y., Robbins J.B., Keith J.M. (2009). Preparation, characterization, and immunogenicity in mice of a recombinant influenza H5 hemagglutinin vaccine against the avian H5N1 A/Vietnam/1203/2004 influenza virus. Vaccine.

[17] Schwarzer J., Rapp E., Hennig R., Genzel Y., Jordan I., Sandig V., Reichl U. (2009). Glycan analysis in cell culture-based influenza vaccine production: Influence of host cell line and virus strain on the glycosylation pattern of viral hemagglutinin. Vaccine.

[18] Fedson D.S. (2008). New technologies for meeting the global demand for pandemic influenza vaccines. Biologicals.

[19] Yap Y.K., Smith D.R. (2010). Strategies for the plant-based expression of dengue subunit vaccines. Biotechnol. Appl. Biochem..

[20] Greenough T.C., Babcock G.J., Roberts A., Hernandez H.J., Thomas W.D., Coccia J.A., Graziano R.F., Srinivasan M., Lowy I., Finberg R.W. (2005). Development and characterization of a severe acute respiratory syndrome associated coronavirus-neutralizing human monoclonal antibody that provides effective immunoprophylaxis in mice. J. Infect. Dis..

[21] Keller M.A., Stiehm E.R. (2000). Passive immunity in prevention and treatment of infectious diseases. Clin. Microbiol. Rev..

[22] Skehel J.J., Wiley D.C. (2000). Receptor binding and membrane fusion in virus entry: The influenza hemagglutinin. Annu. Rev. Biochem..

[23] Gocnik M., Fislova T., Sladkova T., Mucha V., Kostolansky F., Vareckova E. (2007). Antibodies specific to the HA2 glycopolypeptide of influenza A virus haemagglutinin with fusion-inhibition activity contribute to the protection of mice against lethal infection. J. Gen. Virol..

[24] Tumpey T.M., Renshaw M., Clements J.D., Katz J.M. (2001). Mucosal delivery of inactivated influenza vaccine induces B-cell-dependent heterosubtypic cross-protection against lethal influenza A H5N1 virus infection. J. Virol..

[25] Rockman S., Brown L.E., Barr I.G., Gilbertson B., Lowther S., Kachurin A., Kachurina O., Klippel J., Bodle J., Pearse M. (2013). Neuraminidase-inhibiting antibody is a correlate of cross-protection against lethal H5N1 influenza virus in ferrets immunized with seasonal influenza vaccine. J. Virol..

[26] Du L., Jin L., Zhao G., Sun S., Li J., Yu H., Li Y., Zheng B.J., Liddington R.C., Zhou Y. (2013). Identification and structural characterization of a broadly neutralizing antibody targeting a novel conserved epitope on the influenza virus H5N1 hemagglutinin. J. Virol..

[27] Chen Y., Qin K., Wu W.L., Li G., Zhang J., Du H., Ng M.H., Shih J.W., Peiris J.S., Guan Y. (2009). Broad cross-protection against H5N1 avian influenza virus infection by means of monoclonal antibodies that map to conserved viral epitopes. J. Infect. Dis..

[28] Stenler S., Lundin K.E., Hansen L., Petkov S., Mozafari N., Isaguliants M., Blomberg P., Smith C.I.E., Goldenberg D.M., Chang C.H. (2017). Immunization with HIV-1 envelope T20-encoding DNA vaccines elicits cross-clade neutralizing antibody responses. Hum. Vaccin Immunother..

[29] Suradhat S., Wongyanin P., Kesdangsakonwut S., Teankum K., Lumyai M., Triyarach S., Thanawongnuwech R. (2015). A novel DNA vaccine for reduction of PRRSV-induced negative immunomodulatory effects: A proof of concept. Vaccine.

[30] Wang B., Boyer J., Srikantan V., Ugen K., Gilbert L., Phan C., Dang K., Merva M., Agadjanyan M.G., Newman M. (1995). Induction of humoral and cellular immune responses to the human immunodeficiency type 1 virus in nonhuman primates by in vivo DNA inoculation. Virology.

[31] Liu S., Wang S., Lu S. (2016). DNA immunization as a technology platform for monoclonal antibody induction. Emerg. Microbes Infect..

[32] Reed L.J., Muench H. (1938). A simple method for estimating fifty percent endpoints. Am. J. Hyg..

[33] Liu M., Chen X.D., Wang Y., Zhang Y., Li Y.F., Wang Y.F., Shen N., Chen H.L. (2010). Characterization of monoclonal antibodies against Muscovy duck reovirus σB protein. Virol. J..

[34] Yin X.C., Zhang S.M., Gao Y.L., Li J.Z., Tan S.Y., Liu H.Y., Wu X.Y., Chen Y.H., Liu M., Zhang Y. (2012). Characterization of monoclonal antibodies 3 against waterfowl parvoviruses VP3 protein. Virol. J..

[35] Bai X.F., Shaozhou W.L., Zhang Q.S., Li C.X., Qiu N., Meng R.Z., Liu M., Zhang Y. (2015). Characterization of monoclonal antibodies against duck Tembusu virus E protein: An antigen-capture ELISA for the detection of Tembusu virus infection. Arch. Virol..

[36] Liu C.G., Liu M., Liu F., Liu D.F., Zhang Y., Pan W.Q., Chen H., Wan C.H., Sun E.C., Li H.T. (2011). Evaluation of several adjuvants in avian influenza vaccine to chickens and ducks. Virol. J..

[37] Liu M., Liu C.G., Zhang Y., Shi W.L., Wang W., Liu Y.Y. (2012). Efficacy of a high-yield attenuated vaccine strain wholly derived from avian influenza viruses by use of reverse genetics. Vet. Microbiol..

[38] Casadevall A., Dadachova E., Pirofski L.A. (2004). Passive antibody therapy for infectious diseases. Nat. Rev. Microbiol..

